# Histotripsy Ablation Alters the Tumor Microenvironment and Promotes Immune System Activation in a Subcutaneous Model of Pancreatic Cancer

**DOI:** 10.1109/TUFFC.2021.3078094

**Published:** 2021-08-27

**Authors:** Alissa Hendricks-Wenger, Jacqueline Sereno, Jessica Gannon, Allison Zeher, Rebecca M. Brock, Natalie Beitel-White, Alexander Simon, Rafael V. Davalos, Sheryl Coutermarsh-Ott, Eli Vlaisavljevich, Irving Coy Allen

**Affiliations:** Department of Biomedical Sciences and Pathobiology, Virginia-Maryland College of Veterinary Medicine, Blacksburg, VA 24061 USA; Department of Biomedical Engineering and Mechanics, Virginia Tech, Blacksburg, VA 24061 USA; Graduate Program in Translational Biology, Medicine and Health, Virginia Tech, Roanoke, VA 24016 USA.; Department of Biomedical Sciences and Pathobiology, Virginia-Maryland College of Veterinary Medicine, Blacksburg, VA 24061 USA.; Department of Biomedical Engineering and Mechanics, Virginia Tech, Blacksburg, VA 24061 USA.; Department of Biomedical Sciences and Pathobiology, Virginia-Maryland College of Veterinary Medicine, Blacksburg, VA 24061 USA.; Department of Biomedical Sciences and Pathobiology, Virginia-Maryland College of Veterinary Medicine, Blacksburg, VA 24061 USA; Graduate Program in Translational Biology, Medicine and Health, Virginia Tech, Roanoke, VA 24016 USA.; Department of Biomedical Engineering and Mechanics, Virginia Tech, Blacksburg, VA 24061 USA.; Department of Biomedical Engineering and Mechanics, Virginia Tech, Blacksburg, VA 24061 USA.; Department of Biomedical Engineering and Mechanics, Virginia Tech, Blacksburg, VA 24061 USA; Center for Engineered Health, Institute for Critical Technology and Applied Sciences, Virginia Tech, Blacksburg, VA 24061 USA.; Department of Biomedical Sciences and Pathobiology, Virginia-Maryland College of Veterinary Medicine, Blacksburg, VA 24061 USA.; Department of Biomedical Engineering and Mechanics, Virginia Tech, Blacksburg, VA 24061 USA; Graduate Program in Translational Biology, Medicine and Health, Virginia Tech, Roanoke, VA USA; Center for Engineered Health, Institute for Critical Technology and Applied Sciences, Virginia Tech, Blacksburg, VA 24061 USA.; Department of Biomedical Sciences and Pathobiology, Virginia-Maryland College of Veterinary Medicine, Blacksburg, VA 24061 USA; Graduate Program in Translational Biology, Medicine and Health, Virginia Tech, Roanoke, VA USA; Center for Engineered Health, Institute for Critical Technology and Applied Sciences, Virginia Tech, Blacksburg, VA 24061 USA; Department of Basic Science Education, Virginia Tech Carilion School of Medicine, Roanoke, VA 24016 USA.

**Keywords:** Biological effects & dosimetry, therapeutics

## Abstract

Pancreatic cancer is a significant cause of cancer-related deaths in the United States with an abysmal five-year overall survival rate that is under 9%. Reasons for this mortality include the lack of late-stage treatment options and the immunosuppressive tumor microenvironment. Histotripsy is an ultrasound-guided, noninvasive, nonthermal tumor ablation therapy that mechanically lyses targeted cells. To study the effects of histotripsy on pancreatic cancer, we utilized an *in vitro* model of pancreatic adenocarcinoma and compared the release of potential antigens following histotripsy treatment to other ablation modalities. Histotripsy was found to release immune-stimulating molecules at magnitudes similar to other nonthermal ablation modalities and superior to thermal ablation modalities, which corresponded to increased innate immune system activation *in vivo*. In subsequent *in vivo* studies, murine Pan02 tumors were grown in mice and treated with histotripsy. Flow cytometry and rtPCR were used to determine changes in the tumor microenvironment over time compared to untreated animals. In mice with pancreatic tumors, we observed significantly increased tumor-progression-freeand general survival, with increased activation of the innate immune system 24 h posttreatment and decreased tumor-associated immune cell populations within 14 days of treatment. This study demonstrates the feasibility of using histotripsy for pancreatic cancer ablation and provides mechanistic insight into the initial innate immune system activation following treatment. Further work is needed to establish the mechanisms behind the immunomodulation of the tumor microenvironment and immune effects.

## I. Introduction

PANCREATIC cancer is the fourth leading cause of cancer-related deaths, with a 9% survival rate due to its late diagnosis and lack of curative treatment options [1], [2]. Standard treatment options are limited and include surgery, chemotherapy, and radiation [3]. Only 20% of patients have tumors that can be surgically removed, and of those patients, the cure rate is less than 25% [4]. Ablation procedures can act as a replacement for, or as an adjuvant to, surgery. The most commonly used ablation modalities for treating cancers in abdominal organs are radio frequency ablation (RFA), microwave ablation, high-intensity focused ultrasound (HIFU), and cryoablation. RFA is a thermal, minimally invasive ablation modality that utilizes high-frequency alternating currents to thermally induce thermal necrosis [5]. Microwave ablation, also thermal and minimally invasive, induces cell death through electromagnetic microwaves that produce friction and heat [6]. HIFU ablates cells by depositing ultrasound energy at a focal point to rapidly increase tissue temperature [7]. Cryoablation results in tumor cell destruction through icecrystal formation as a result of liquid nitrogen or argon gas delivered to the tissue and does not result in protein denaturation, which has led to increased reports of immunological effects compared to RFA and microwave ablation [5]. The thermal ablation modalities, although efficacious in treating certain malignancies, have not yet been ubiquitously accepted into clinics for pancreatic cancer therapy due to the risk of thermal damage to healthy pancreatic tissue, vasculature, and other critical structures.

Recent advancements have established nonthermal therapies that have the potential to treat pancreatic cancer. For example, irreversible electroporation (IRE) utilizes short, high-voltage electrical pulses that nonthermally open micropores in cell plasma membranes, inducing cell death [8]. Clinical trials have shown that IRE can ablate pancreatic tumors without damaging nearby critical structures and have had dramatic effects on patient survival that may be due to the induction of immunomodulatory mechanisms [9]-[14]. Given IRE’s controlled cell death mechanisms, the procedure has been found to release immunostimulatory molecules and lead to a more immunologically active tumor microenvironment after treatment [15]. Furthermore, recent studies comparing the thermal ablation modalities, cryoablation, and IRE have found that the nonthermal modalities are more potent at stimulating an antitumor microenvironment [16]. These procedures still involve surgical incisions that increase the possibility of surgery-related injury or infection.

To address the clinical limitations of IRE and other ablation modalities, new focused ultrasound ablation methods have been studied as a completely noninvasive, nonthermal alternative. Histotripsy is a nonthermal, nonionizing, imageguided ablation modality that uses focused ultrasound to initiate acoustic cavitation, which leads to the lysis of cells contained in the targeted area [17], [18]. Ablation of internal targets with histotripsy is effective with little to no off-target effects [19]-[21]. Early studies with histotripsy ablation of melanoma, hepatocellular carcinoma, renal cell carcinomas, colorectal carcinomas, and neuroblastomas established that there is an activation of local, cellular, and systemic immune responses [22]-[27].

In this study, we assess the ability of histotripsy to ablate subcutaneous pancreatic tumors and determine the immunological changes within the treated tumors over time. First, we compared the release of damage-associated molecular patterns (DAMPs) known to activate the innate immune system and neoantigen generation following histotripsy against other tumor ablation modalities. We extended these *in vitro* findings using the *in vivo* Pan02 murine pancreatic cancer model. This mouse model was chosen given its well-characterized progression and described immune effects to cancer therapies [15], [28], [29]. Using this model, we demonstrate effective tumor ablation and proinflammatory changes in the tumor microenvironment and identify critical immune signaling mechanisms associated with histotripsy treatment.

## II. Procedures

### A. Tumor Injections and Monitoring

All *in vivo* experiments were conducted under institutional IACUC approval and following the NIH Guide for the Care and Use of Laboratory Animals. For these studies, male and female mice were equally utilized in the 7–10-week age range. Once the entire cohort of C57/Bl6 mice reached a minimum weight of 20 g, 100 *μ*L of Pan02 cells (DTP and DCTD tumor repository) at a concentration of 6.0 × 10^7^ cells/mL of Matrigel (Corning) were injected into the right flank of the mice that were anesthetized with vaporized isoflurane (1.5 L/min oxygen flow with 1%–3% isoflurane). Control animals were injected with the same amount of Matrigel that did not contain Pan02 cells. The mice were then monitored three times a week until the end of the study. Tumor diameters were measured with calipers and calculated as the square root of two perpendicular measurements, as previously described [24]. The weights and tumor sizes were recorded along with the general health of the mice.

### B. Histotripsy Setup

**Fig. 1.**
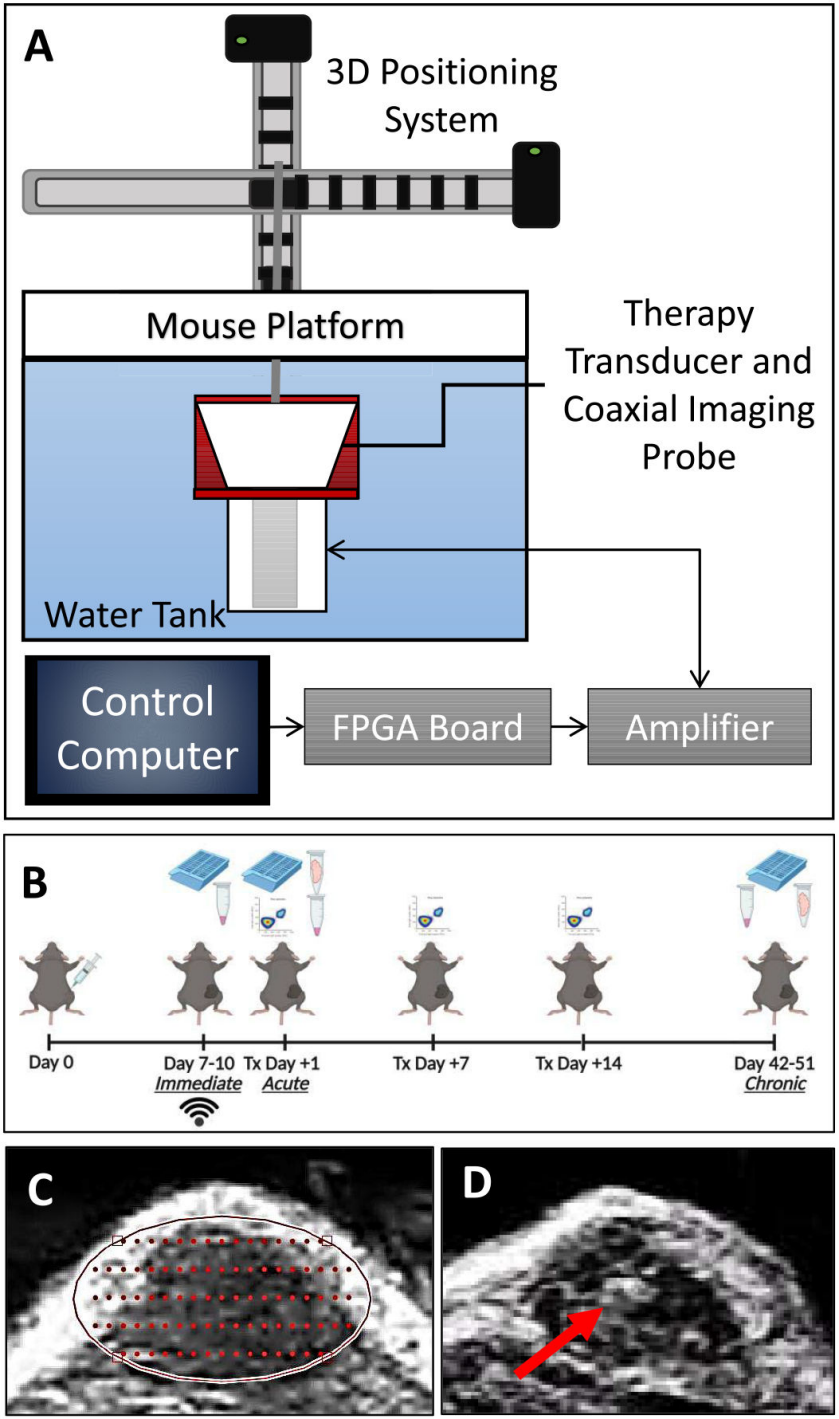
*In vivo* experimental setup. (a) Murine histotripsy experiments were conducted using a 1-MHz transducer. (b) Timing for treatment, euthanasia, and data collection were set as diagramed. Days with histology collection are noted by cassettes, flash-frozen tumors noted by tubes with tumors, and serum noted by tubes with serum, and flow cytometry is noted by flow charts. Tumors located with ultrasound imaging were used for (c) planning automated ablation disks and (d) raster scan plots. (e) Therapy was guided by coaxially aligned ultrasound imaging. The red arrow indicates a bubble cloud that was generated during treatment.

*In vivo* studies used a custom 1-MHz, eight-element small animal histotripsy transducer with a geometric focus of 36 mm, an aperture size of 52.7 mm, and an f-number of 0.68. The full-width at half-maximum (FWHM) dimensions at a geometric focus of this transducer were 0.98, 0.93, and 3.9 mm in transverse, elevational, and axial, respectively. The transducer was driven via a custom high-voltage pulser designed to generate short therapy pulses of <2 cycles controlled by a field-programmable gate array (FPGA) board (Altera DE0-Nano Terasic Technology, Dover, DE, USA) programed for histotripsy therapy pulsing. The transducer was positioned in a tank of degassed water heated to 37 + 4 °C beneath a custom-designed mouse surgical stage [see Fig. 1(a)] and attached to a computer-guided 3-D positioning system with a 0.05-mm motor resolution to control the automated volumetric treatments. A linear ultrasound imaging probe with a frequency range of 10–18 MHz (L18-10L30H-4, Telemed, Lithuania, EU) was coaxially aligned inside the transducer for treatment guidance and monitoring [20], [24]. The transducer was powered by a high-voltage dc power supply (GENH750W, TDK-Lambda), and the system was controlled using a custom user interface operated through MATLAB (MathWorks).

### C. In Vitro Ablation Treatment Parameters

**Fig. 2.**
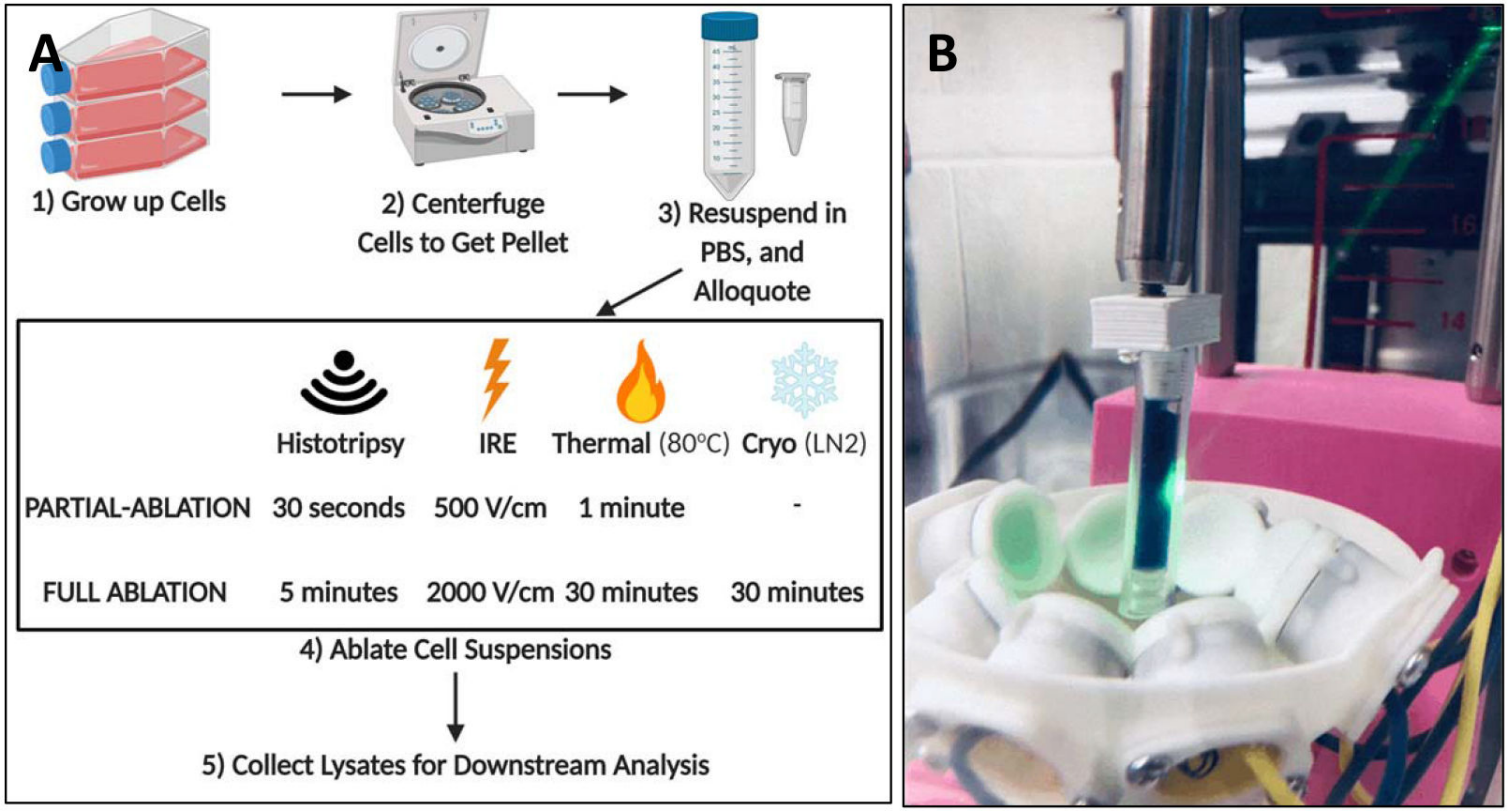
(a) Treatment flow and dosages for various ablation modalities. (b) Histotripsy experiments on cell suspensions were conducted using a 1-MHz transducer.

Pan02 cells transfected with a plasmid that produces the influenza antigen hemagglutinin (Pan02-HA) were used for these *in vitro* experiments. HA is a common surrogate used in tumor-specific antigen studies. Pan02-HA cells were ablated in four treatments and two levels of ablation [see Fig. 2(a)]. Cells were collected and centrifuged to pellet and were resuspended in PBS at a concentration of 10 × 10^6^ cells/mL; 1 mL of cell suspension was used for each treatment. All samples were kept on ice until treatment. For all treatments, the partial ablation dosages were determined to be a successful ablation with >30% viability, and the full ablation dosages had <10% viability for all samples. For each ablation modality, the prefix “f-” is added before the modality name within the text to refer to the full ablation, and “*p*-” is added to refer to the partial ablation. For example, f-histotripsy is full ablation histotripsy and *p*-Histotripsy is partial ablation histotripsy.

Histotripsy treatments were done in a custom holder [see Fig. 2(b)], utilizing the same 1-MHz transducer from the *in vivo* work at a PRF of 250 Hz at a single spot for 0.5 or 5 min. The movement of the transducer during treatment was not necessary given the circulation of the cell suspension caused by histotripsy that was observed during treatment and in previous studies [30]. For cryoablation, cells were placed in liquid nitrogen (approximately −160 °C) for 30 min. No lower dose was used given that any drop to therapeutically low temperatures (−20 °C to −190 °C) resulted in high levels of cell death [31]. Thermal ablation consisted of cells being kept at 80 °C on a heating block for 1 or 30 min. The protocols for cryoablation and thermal ablation were based upon a previous study [16]. For samples not included in the analysis, the temperatures of the thermally treated samples were confirmed with a thermometer to reach 45 °C, minimum temperature to be considered thermal ablation, within the first half of a minute and 80 °C by 3 min [32]. Samples frozen with liquid nitrogen were assumed to surpass the therapeutic temperature threshold. Given the extensive prior studies showing no temperature change with IRE at the prescribed dosages or histotripsy, no temperature measurements were done for these therapies [17], [33]-[35]. For experimental sham, untreated control samples of cells were prepared and handled identically to treated samples but, instead of receiving treatment, remained on wet ice during the treatment of other cells.

For IRE treatments, cells were suspended in a sucrose solution, described previously to improve the quality of electrical transduction [36], and placed into 4 mm cuvettes. A generator (BTX ECM 830, Harvard Apparatus, Holliston, MA, USA) was used to apply 100 pulses with widths of 100 *μ*s at a frequency of 1 Hz and an electric field of either 500 or 2000 V/cm. Cells were then cultured for 24 h before supernatant collection to allow for the controlled cell death of IRE to take place [15]. Additional controls were collected at this point to accommodate for the extra downstream processing of the IRE samples. In these cases, the media that was used to culture the IRE samples overnight was also run through BCA and nanodrop. The resulting values from the controls were subtracted from the IRE samples to accommodate for the excess signaling caused by the media in culturing.

Viability for all modalities was determined shortly after treatment with standard Trypan Blue counting with a 1:40 dilution due to the high concentration of cells and calculated as a percentage of cells remaining viable after treatment. Postablation samples were centrifuged at 1000xg for 5 min, and supernatants were collected. Supernatants for cryoablation, thermal ablation, and histotripsy were collected immediately after treatment and IRE 24 h after incubation at 37 ^°^C.

### D. In Vivo Histotripsy Treatment

Mice were treated when the average tumor diameter of the cohort was approximately 0.6 cm, to ensure that the smallest tumors were large enough for targeting [see Fig. 1(b)]. Animals were euthanized 24 h after treatment, referred to as the acute group (*n* = 7 treated, *n* = 7 untreated controls, and *n* = 3 tumor-free untreated controls), or at survival time points, referred to as the chronic group (*n* = 7 treated, *n* = 7 untreated controls, and *n* = 3 tumor-free untreated controls) (see Table I). Euthanasia of chronic group animals, the point determined as their general survival, was determined as either: 1) when clinical health evaluations found deleterious symptoms including hunching, irregular respiratory rate and rhythm, decreased alertness and socialization, or poor extremity utilization or 2) when the tumor diameter exceeded 1.4 cm. At necropsy, the immediate group (*n* = 3) mice that were treated and untreated (see Table I) were euthanized, and tumors were formalin fixed for histopathology to determine the efficacy of treatment. For the acute and chronic group mice, serum was collected, and the tumors were sectioned with a portion formalin-fixed for histopathology and another portion flash-frozen for mRNA analysis [see Fig. 1(b)].

Prior to each treatment, each mouse had fur removed over the tumors using the depilatory cream Nair (Naircare, Ewing, NJ, USA). Mice were anesthetized with vaporized 2%–4% isoflurane with an oxygen flow rate of 1.5 L/min. The mice were then placed on the subject stage with their tumor submerged in the degassed water in the subject stage’s window. The tumor was located using the ultrasound imaging probe that was coaxially aligned to the histotripsy transducer and then targeted using an automated volumetric ablation algorithm that controlled the treatment following manual targeting.

For each tumor, a 3-D ellipsoidal volume was targeted using conservative margins of approximately 0.5–2 mm from the skin and underlying tissues (muscle, intestines, and so on). Since our automated treatment is a perfect ellipsoid and tumors are not, there was some small amount of variation between subjects. Using these margins, we intentionally targeted a partial ablation of approximately 60%–75% of the tumor volume. This volume consisted of multiple, concentric 2-D elliptical slices. Each treatment slice was separated 0.75 mm apart. Within each slice, the area was populated with grid points separated 0.75 mm apart in the transverse direction and 1.25 mm apart in the axial direction [see Fig. 1(c)]. At each treatment point, histotripsy was applied at a pulse repetition frequency (PRF) of 250 Hz and a dwell time of 1 s, consequently sending 250 pulses to each point. For each slice, the automated treatment first generated a histotripsy bubble cloud at the center point of the slice and then scanned in a raster pattern to cover one half of the slice area. The transducer was then returned to the center of the ellipse and scanned in a raster pattern to cover the other half of the slice. Once a slice was scanned through completely, the system proceeds to the next slice, which is repeated until the entire ellipsoidal volume was treated. Throughout treatment, ultrasound guidance confirmed the location of the bubble cloud for the duration of the volumetric ablation [see Fig. 1(d)]. After treatment, the tumor volume was again imaged with ultrasound imaging in order to assess for tissue ablation.

### E. Determining DNA Release and Quality

Each collected sample for each treatment group (*n* = 5 for the no treatment group and *n* = 9-11/treatment group) was analyzed with a nanodrop drop, and the DNA concentrations (ng/*μ*L) and the 260/280 absorbance ratios were recorded. Samples were then run on an ethidium bromide gel to visualize DNA strand sizes present in the samples utilizing a 100bp HyperLadder (Bioline) and following standard protocol.

### F. Determining Protein Release and Quality

The protein released was quantified using a BCA assay (Thermo Scientific) following the manufacturer’s protocol. Western blots were run using premade gels (Thermo Scientific) following standard protocols with 5 *μ*g of protein/sample and transferred with the iBlot 2 Dry Blotting System (Thermo Scientific). HA antigen release was determined using an HA antibody (Cell Signaling) as per the manufacturer’s protocols. The protein’s band area was quantified using iBright Analysis Software (*n* = 5/treatment group).

### G. Histopathology

Tissues were harvested from animals and fixed in 10% formalin. Paraffin-embedded formalin-fixed tissues were stained with hematoxylin and eosin (H&E) following standard protocols. Evaluations were performed by trained individuals and independently verified by a blinded, board-certified veterinary pathologist (S.C.O.).

### H. Profiling Gene Expression and Pathway Analysis

Using tumors flash-frozen from *in vivo* experiments, total RNAs are isolated from tumor samples using the RNeasy Mini Kit (250) (Qiagen), where the manufacturer’s standard protocol was followed. RNA levels were quantified with a nanodrop, converted to cDNA, and were then pooled (*n* = 7/tumor group and *n* = 3/control group). Pooled cDNA was placed into the commercially available pathway-focused array “Cancer Inflammation and Immunity Crosstalk” (SuperArrayTM platform; Qiagen) following the manufacture’s protocol (1/pooled groups). Fold change was determined using standard ΔΔCT calculations. Gene expression was analyzed using integrated pathway analysis (IPA, Qiagen) software to model changes in complex pathways [36].

### I. Flow Cytometry Panel Staining

For immune cell profiling, additional mice were injected with tumors and taken down one, seven, and 14 days after treatment [see Fig. 1(b)] with an *n* = 4 per treated and untreated control group at each time point (see Table I).

After removal of the tumor, avoiding skin and fur, the tumor was placed into 8 mL of cold RPMI. Even though Pan02 tumors have microvasculature [37] because they are not very bloody due to total blood removal via heart stick, we did not perfuse the tissue before harvest or processing. The tissue was then mechanically digested and strained into a 50-mL conical tube. After centrifuging at 300xg for 10 min at 4 °C, the supernatant was discarded and the pellet resuspended in 10 mL of RPMI and was plated onto a 96-well V-bottom plate at a density of 1.0 × 10^6^ cells/well. A 1:200 dilution of FACS buffer, sterile PBS with 2% FBS, 0.1% sodium azide, and anti-CD16/32 was added to the plate at a concentration of 50 *μ*L/well. Antibodies were diluted with FACS buffer and added directly to the wells. The following antibodies were used: anti-CD8 SuperBright 645, anti-CD45 SuperBright 645, anti-CD11c APC, anti-CD45 PE, anti-F4/80 FITC, anti-CD4 PE.Cy5, anti-CD8 A488, anti-CD3 APC, anti-Ly6C APC Cy5, anti-Ly6G PE, and anti-FOXP3 PerCP Cy. Cells were washed with PBS and evaluated with FACS (BD Biosciences). Gating for specific immune cell populations is listed in Table II. It should be noted that the population “granulocytes” is likely to contain both granulocytic myeloid-derived suppresser cells and neutrophils [38], [39]. In addition, by gating macrophages as both CD11c and F4/80 that we are most likely focusing on a subpopulation of macrophages, more extensive panels would be necessary to confirm the ratio of subpopulations.

### J. HMGB1 Serum ELISA

Serum levels of high mobility group protein (HMGB1) in acute and survival group mice were determined utilizing an ELISA assay kit (ABclonal) following the manufactures suggested protocol.

### K. Statistics

Data were analyzed using GraphPad Prism, version 8. Statistical significance was defined as *p* ≤ 0.05, where values were not significant, but *p* < 0.20, where the value is noted in the text. All data are represented as the mean ± SEM. A student’s two-tailed t-test was used when comparing two experimental groups. When many t-tests were performed for one graph, group letter designations were used. Lowercase letters on top of bars indicate significance; bars with the sample letter designation are not significant, while those that do not share a letter are significant (*p* < 0.05). Multiple comparisons were done using one- or two-way ANOVA where appropriate and then followed by the Tukey posttest for multiple pairwise examinations.

## III. Results

### A. Different Ablation Modalities Show Differential Release of Damage Associated Molecular Patterns (DAMPs) and Potential Antigens

**Fig. 3.**
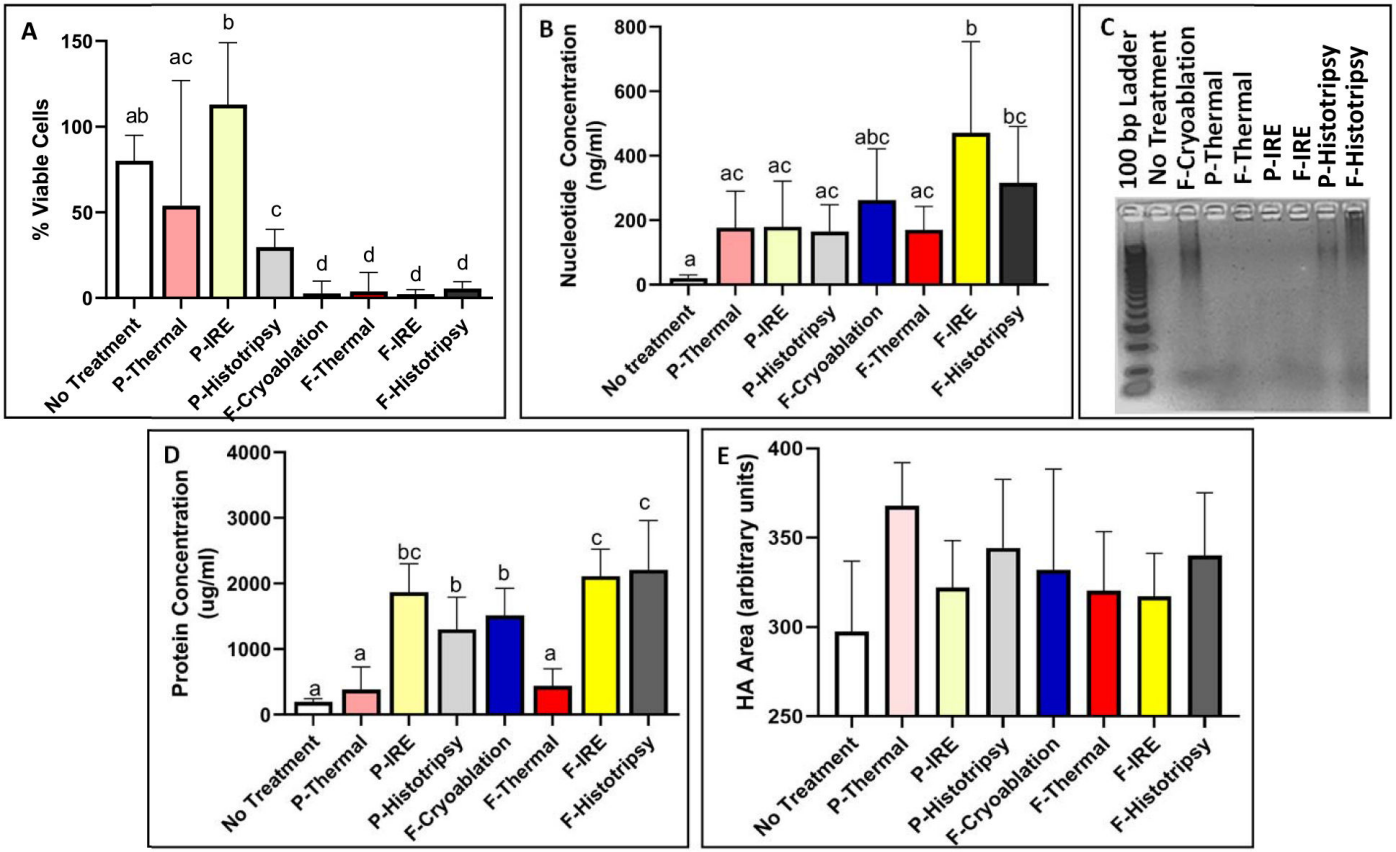
(a) Cell suspension ablations resulted in partial and full ablations. DNA release was (b) quantified and (c) visualized. (d) Protein release was quantified, and (e) relative release of the antigen HA was quantified from western blot bands. Lowercase letters on top of bars indicate significance; bars with the sample letter designation are not significant, while those that do not share a letter are significant (*p* < 0.05).

Pan02-HA cells were treated with thermal ablation, cryoablation, IRE, and histotripsy at full (f-) and partial (*p*-) doses and were chosen based on previous literature [see Fig. 2(a)] [16]. Treatments that lead to less than 10% viability in all samples were considered fully ablated, and those that were greater than 30% in all samples were considered partially ablated [see Fig. 3(a)]. All samples’ lysates were analyzed for peptide and DNA nucleotide release (*n* = 5 for no treatment and *n* = 9-11 for treatment groups).

Extracellular DNA is a DAMP and often correlates with extracellular nuclear proteins, such as HMGB1, which also acts as robust damage signaling. Here, we observed increased levels of DNA for all treatments compared to untreated samples [see Fig. 3(b)]. DNA release was evaluated by nucleotide quantification, which showed f-IRE (470.73 ± 283.34 ng/ml) to have a significantly (*p* < 0.05) higher concentration than most other modalities, excluding f-cryoablation (262.49 ± 159.00 ng/ml) and f-histotripsy (315.71 ± 175.28 ng/ml). P-thermal (177.33 ± 113.15 ng/ml), f-thermal (170.48 ± 72.20 ng/ml), *p*-IRE (179.82 ± 141.98 ng/ml), and *p*-histotripsy (165.03+83.09 ng/ml) were all similar to each other. Only f-IRE and f-histotripsy were significantly greater than the untreated control samples (20.35 ± 10.11 ng/ml). Gel electrophoresis showed that cryoablation and both dosages of histotripsy left large segments of detectable DNA, while untreated, IRE, and thermally ablated samples produced no visible bands [see Fig. 3(c)].

Peptide release, which correlates with potential antigens, was observed to be released significantly in nonthermal ablation modalities [see Fig. 3(d)]. There was no significant difference between *p*-thermal (383.8 ± 345.4 *μ*/ml) or f-thermal (443.3 ± 255.8 *μ*/ml) ablation’s peptide release from samples that were not treated (194.5 ± 50.7 *μ*/ml). On the other hand, f-IRE (2113 ± 409.1 *μ*/ml) and f-histotripsy (2208 ± 751.8 *μ*/ml) were significantly (*p* < 0.05) higher levels of release compared to all other modalities, except for *p*-IRE (1869 ± 431.3 *μ*/ml). F-cryoablation (1512 ± 415.4 *μ*g/ml) and *p*-histotripsy (1300 ± 490.0 *μ*g/ml) were significantly different from the low (significance group “a”) and high release clusters (significance group “c”).

Release of HA was confirmed on western blot for all treated samples, regardless of ablation treatment (see Fig. 1 in the Supplementary Material). The average area of the various treatments HA bands was not significantly different between any therapies compared to each other nor the untreated samples (297.6 ± 39.3 units) [see Fig. 3(e)]. The highest detection of HA was found in *p*-thermal treated samples (367.8 ± 24.25 units), followed by *p*-histotripsy (344.3 ± 38.5 units) and f-histotripsy (340.0 ± 35.2 units). F-cryoablation (332.0 ± 56.4 units), f-thermal (320.3 ± 33.1 units), *p*-IRE (322.0 ± 26.4 units), and f-IRE (317.2 ± 24.2 units) were all found to have larger bands than no treatment and smaller than *p*-thermal, *p*-histotripsy, and f-histotripsy.

### B. Histotripsy Is an Effective Tumor Ablation Modality in the Subcutaneous Pan02 Model

**Fig. 4.**
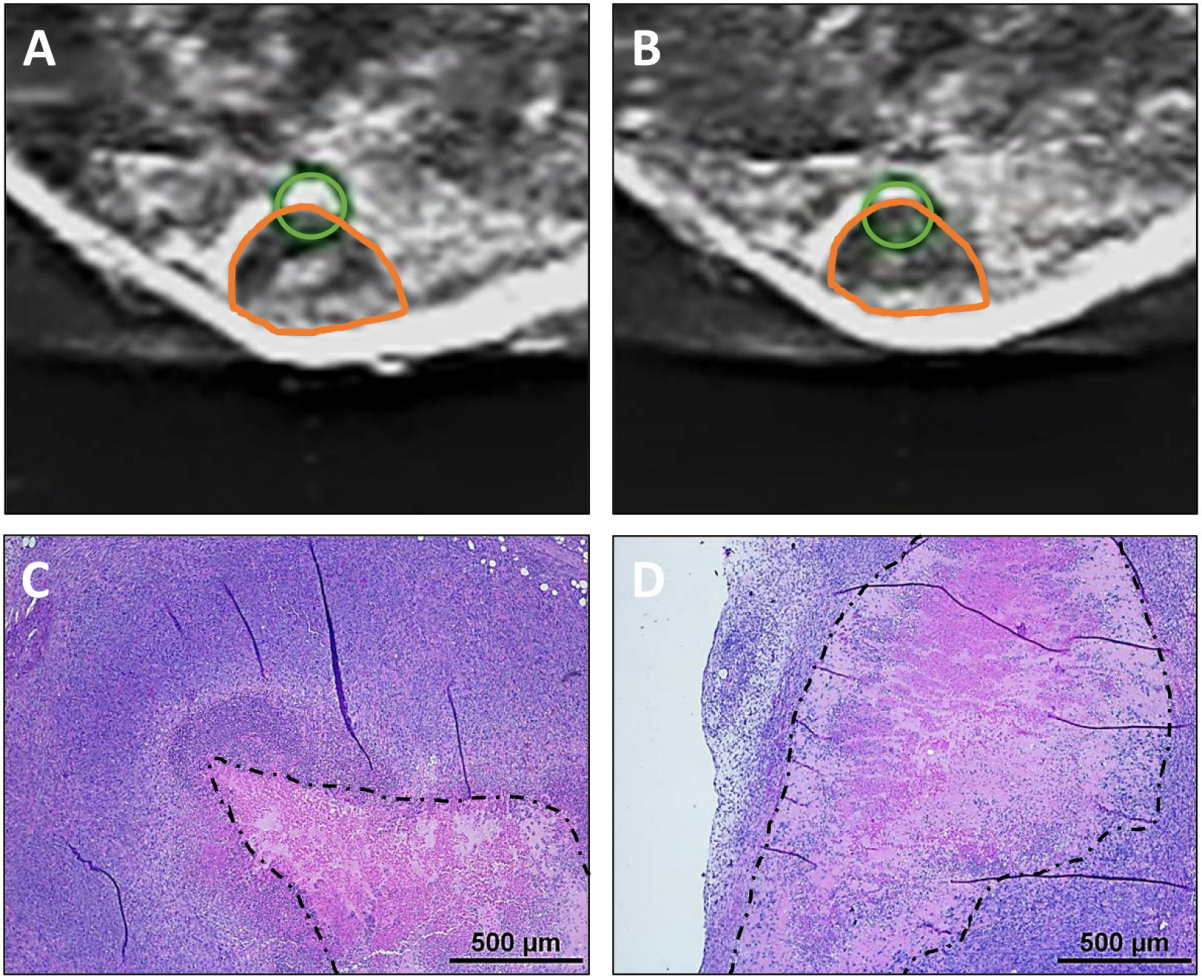
Ultrasound images of tumor (a) before and (b) after treatment, exhibiting more hypoechoic central region posthistotripsy. Orange shapes on ultrasound indicate the location of tumors. Green circles indicate the location of bubble cloud determined in water prior to treatment and were utilized for treatment planning. Histology to tumors (c) without and (d) with treatment shows decreased cellular detail after treatment. The dotted black line outlines the necrotic core of the tissue. Scale bar on H&E images = 500 *μ*m.

The schematic shows the custom histotripsy rig and automated ablation procedure that was used for *in vivo* treatments (see Fig. 1). Coaxial ultrasound imaging confirmed the presence of a bubble cloud within the tumors during treatment [see Fig. 1(e)]. Mice were treated when tumors reached 0.6 cm in diameter on average and harvested in groups, as depicted in Fig. 1(b). Ablation of tumors was confirmed with ultrasound, with increased hypoechoic regions and histopathology, where a partial ablation of tumors with viable tumor tissue on margins and in islands within the ablation zone was observed (see Fig. 4). On ultrasound images, the center of the treated region had a more notable decrease in ultrasound reflection, while the dermal margin maintained a comparable hyperechocity after treatment compared to before [see Fig. 4(a) and (b)]. This pattern was also noted in histopathology. The center of untreated tumors has a characteristic necrotic core [see Fig. 4(c)], and the treated tumors appeared to have a larger region of cell death that extends nearly to the margins of the tumor [see Fig. 4(d)].

**Fig. 5.**
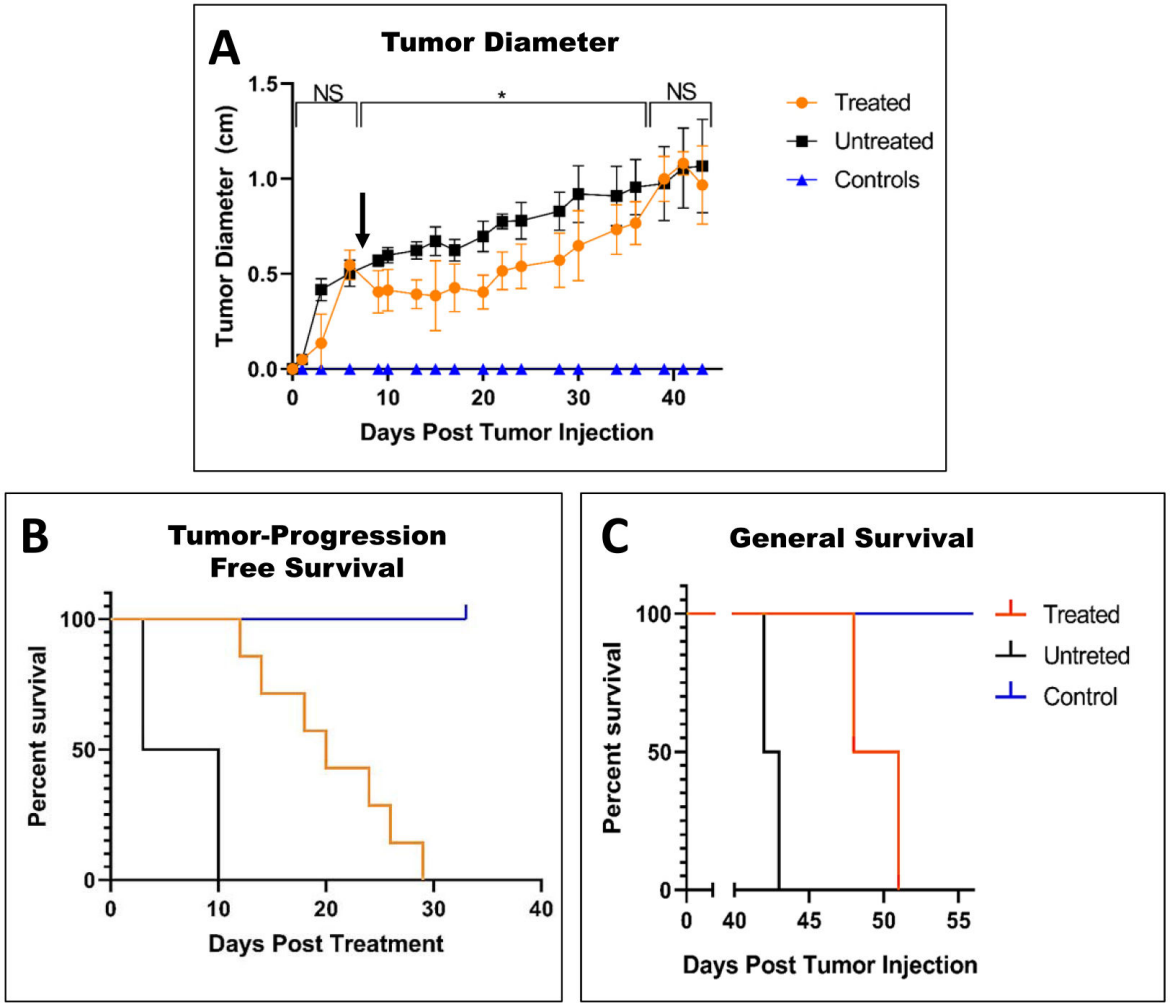
After treatment of tumors on day 8, as indicated by the black arrow, the reduction of tumor volume was indicated by (a) caliper measurements. Changes in health observed in (b) tumor-progression-free survival and (c) general survival.

Calculated tumor diameters decreased for a few days after the partial ablation with histotripsy and maintained size for two weeks before resuming tumor growth [see Fig. 5(a)]. The average tumor size of untreated tumors continued to increase in size at a relatively steady rate [see Fig. 5(a)]. The greatest difference between the treated and untreated groups was reached on day 15, eight days posttreatment when the treated tumors were 43% of the size of the untreated tumors on average. Compared to the untreated mice, histotripsy treatment increased tumor-progression-free survival by 19 days from ten to 29 days posttreatment [see Fig. 5(b)] and general survival by eight days from 43 to 51 days posttreatment [see Fig. 5(c)].

### C. Histotripsy Ablation Results in Increased Acute Cellular Immune Response

**Fig. 6.**
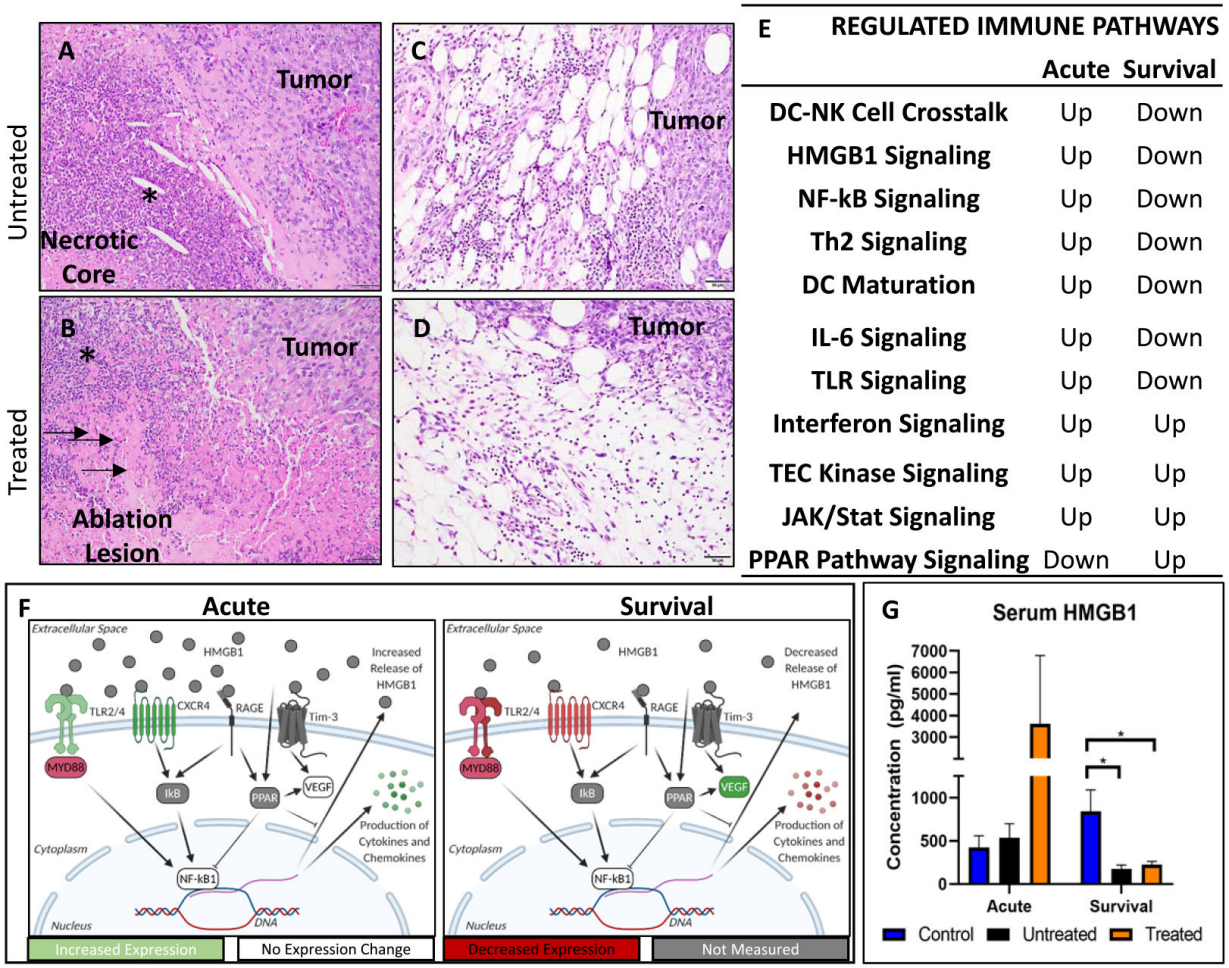
We initially chose to investigate changes in immune cell infiltration following histotripsy treatment by evaluating the tissues microscopically, (a) Untreated tumors exhibit necrotic cores as part of the natural progression of the tumor. These are characterized by predominantly lytic cellular debris but also often contain variable numbers of degenerate and non degenerate neutrophils (asterisk). (b) Treated tumors likewise develop similar foci of necrosis with lytic debris and variable neutrophilic infiltration. In addition, they also exhibit ghost cells which are eosinophilic (pink) remnants of dead cells (arrows) as well as hemorrhage (not shown). In addition, both untreated (c) and treated (d) tumors exhibit a mixture of inflammatory cells at the periphery of the tumor. Microscopically there are no appreciable differences between infiltration of these inflammatory cells between untreated and treated tumors. Analysis of mRNA expression in treated tumors showed regulation of immune pathways (e). Regulated pathways interact with each other and are up regulated in the acute group and downregulated in the chronic group (f). Serum HMBG-1 levels (g) correlate with the mRNA upregulation of HMGB-1 associated pathways.

These tumors even without treatment tend to develop a central area of necrosis as they progress. These necrotic cores are often characterized by accumulations of cell debris with variable numbers of infiltrating degenerate and nondegenerate neutrophils. Otherwise, appreciable numbers of immune cells are relatively absent microscopically throughout the tumor tissue [see Fig. 6(a)]. Treated tumors also exhibited a core of cell death following ablation with variable infiltration by predominantly degenerate neutrophils. However, in addition to cellular debris, these cores also often contain ghost cells and acute hemorrhage [see Fig. 6(b)]. In both treated and untreated tumors, small to moderate numbers of neutrophils, macrophages, lymphocytes, and/or plasma cells are present at the tumor periphery. There is no appreciable difference between the two [see Fig. 6(c) and (d)].

Despite the lack of appreciable differences in immune cell populations microscopically, we investigated immune cell signaling through gene expression. Superarray rtPCR showed that many genes associated with cancer inflammation and immunity crosstalk were significantly regulated after histotripsy treatment (see Table 1 in the Supplementary Material). The IPA analysis comparing treated animals to untreated in acute and survival groups found multiple canonical immune pathways regulated by histotripsy treatment [see Fig. 6(a)]. As a trend, the proinflammatory pathways are upregulated 24-h treatment, but the majority of these pathways become downregulated and were replaced with more anti-inflammatory pathways at survival points. Many of the upregulated pathways (HMGB1, NF-*κ*B, IL-6, and TLR signaling pathways) are interconnected and are self-regulated to decrease in function over time [see Fig. 6(b)]. The schematic in Fig. 5 illustrates the interactions and mechanisms identified by IPA that is upregulated in the acute treatment group and downregulated in the survival group. Some of these proteins and pathways were not directly analyzed but are predicted to be modulated based upon molecules that are upstream and downstream by IPA. Together, these data indicate a significant upregulation of pathways associated with the activation of the innate immune system associated with DAMP signaling.

HMGB1 signaling was identified by IPA and is a potent DAMP. To verify this aspect of our pathway analysis, we evaluated the protein levels of HMGB1 in the serum with and without histotripsy treatment. Consistent with the IPA results, HMGB1 levels were increased in the sera of treated mice at the acute time point compared to untreated and control animals, while both treated and untreated mice saw a significant (*p* < 0.05) decrease in serum HMGB1 levels at survival endpoints [see Fig. 6(c)]. Although there was a notable increase, due to the significantly higher variance (*p* = 0.0032), there was no significance in the average serum HMGB1 level in the treated acute group compared to the untreated and control groups.

### D. Histotripsy Alters Immune Cell Composition in the Tumor Microenvironment

**Fig. 7.**
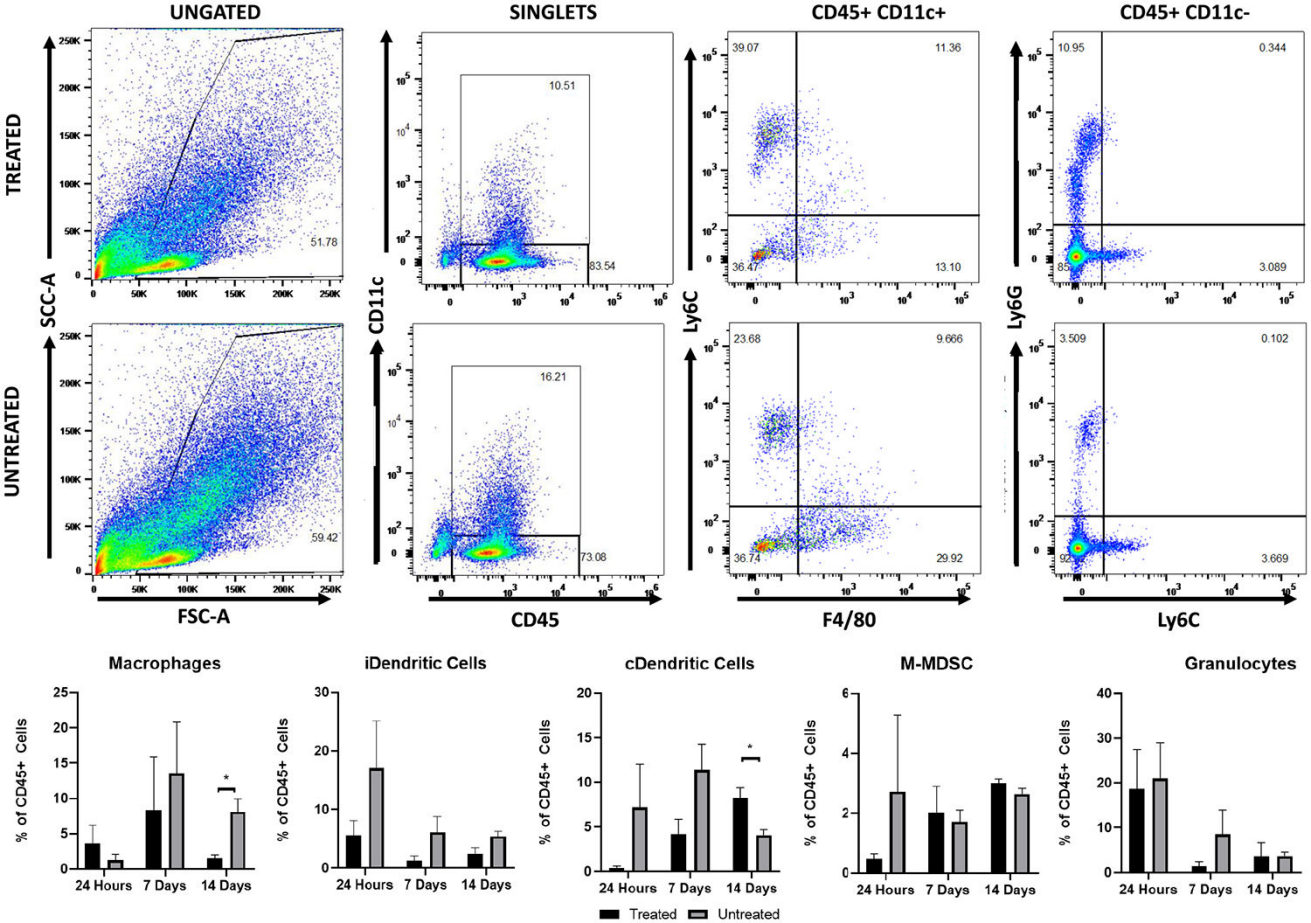
Single-cell suspension from treated and untreated tumors were collected at 24 h and seven and 14 days after treatment and were stained as described for flow cytometry to identify innate immune cells. The percentage of each innate immune cell population analyzed was calculated as part of the total CD45+ cells stained. Example flow cytometry plots from treated and untreated tumors at 14 days after treatment.

**Fig. 8.**
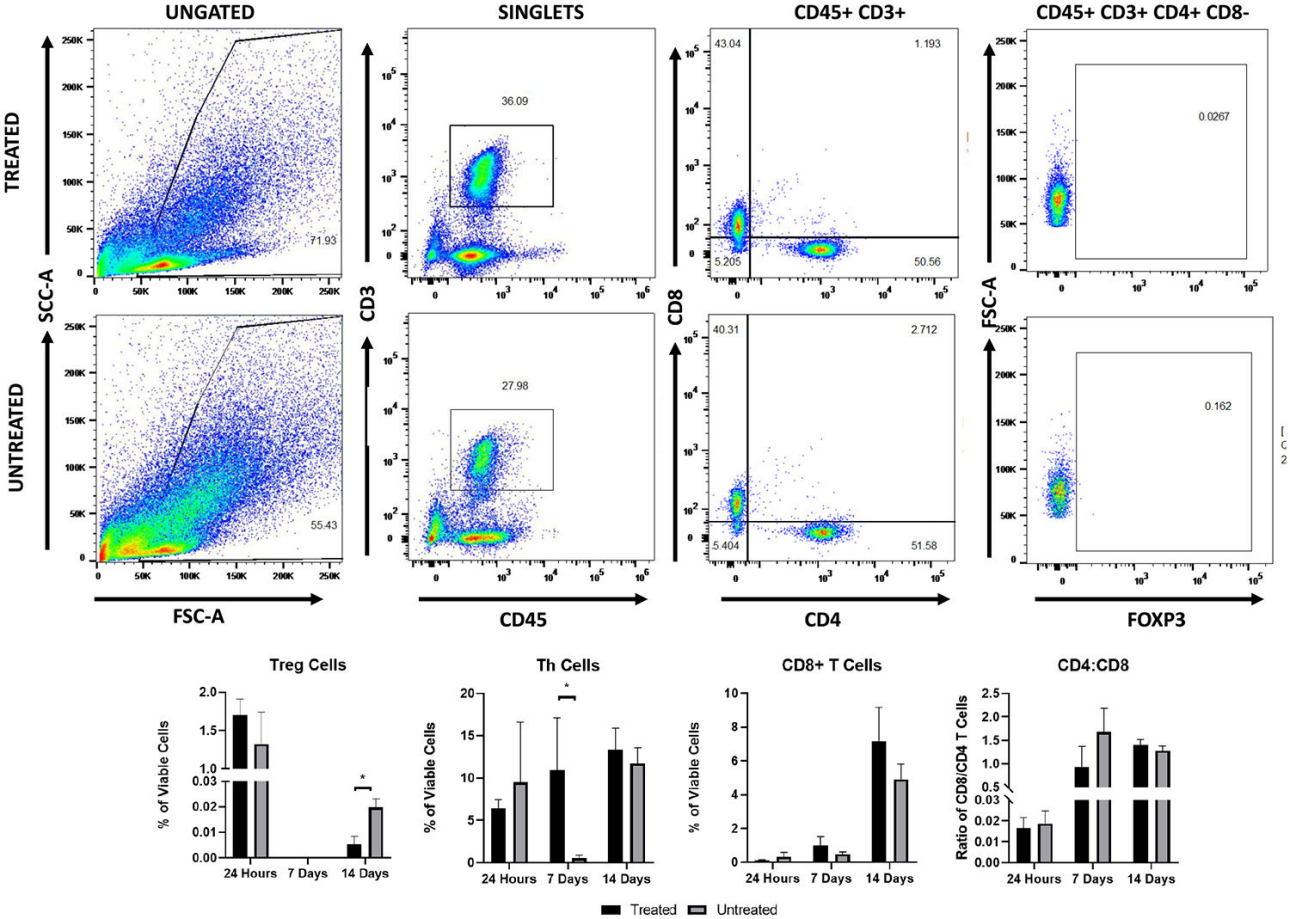
Single-cell suspension from treated and untreated tumors were collected at 24 h and seven and 14 days after treatment and were stained as described for flow cytometry to identify adaptive immune cells. The percentage of each adaptive immune cell population analyzed was calculated as part of the total viable cells stained (gated as singlets). The ratio of CD4+/CD8+ T cells was calculated as a simple ratio. Example flow cytometry plots from treated and untreated tumors at 14 days after treatment.

To quantify changes in immune cell populations within tumors zovertime after histotripsy treatment, tumors were collected 24 h and seven and 14 days after treatment for flow cytometry. Although there were no significant changes at the 24-h time point, there was an appreciable decrease in inflammatory dendritic cells (iDCs, *p* = 0.19), classical dendritic cells (cDCs, *p* = 0.18), granulocytes, Th cells, CD4+ T cells, and CD8+ T cell populations after treatment (see Figs. 6 and 7). The relative reduction of iDCs (*p* = 0.13) and cDCs (*p* = 0.07) continued to day 7 along with a decrease in M-MDSCs; at this point, the remaining cells that appeared reduced at 24 h were more comparable to the untreated (see Fig. 7). At 14 days after treatment, macrophages and Treg cells were found to be significantly reduced, while cDCs were found to be significantly increased, and neutrophils (*p* = 0.19) notably increased in treated tumors compared to the untreated (see Fig. 7 and 8). While there were no changes in the ratios of CD4+/CD8+ T cells in the treated tumors compared to the untreated, there were significant increases in CD4+ and a notable increase in CD8+ T cells within the treated tumors at seven days after treatment (see Fig. 8).

## IV. Discussion

This study investigated the treatment of pancreatic tumors with histotripsy and the resultant immunological responses, with a focus on the innate immune system. To optimize our immune system assessments, we established conservative margins with the expectation that some tumors would not be treated. This allowed cells within the untreated margins to respond to the effects of our treatment and provided specimens for subsequent analysis. For *in vivo* ablations, histotripsy treatment was confirmed via bubble-cloud formation, while H&E showed complete ablation within targeted regions. Overall, we achieved a partial ablation of all treated tumors. There was an average reduction of 43% in the tumor diameters of the remaining tumor tissue after treatment, comparable to tumor retardation compared to previous histotripsy subcutaneous tumor treatments [20], [23], [24]. Based upon the fully ablated tissue found within the targeted regions on H&E, the remaining tissue can be assumed to have been untreated based upon the margins set. This led to tumors that were fully ablated in certain regions and completely untreated in others. More complete ablation of tumors with histotripsy has been achieved in *de novo* and *in situ* studies where margins include surrounding healthy tissues, but these tumors still recurred [40]-[43]. In this study, the progression of tumor growth was improved with treatment [see Fig. 5(b)]. However, in line with previous studies, tumor recurrence occurred and minimized the improvement of the general survival for animals that received treatment [see Fig. 5(c)].

The release of DAMPs is a consistent feature of focal ablation modalities, and the established modalities have been compared to each other in previous work [16]. Histotripsy has not been previously, directly compared to nonultrasonic ablation modalities. The effects of histotripsy compared to other ablation modalities for producing immune-stimulating molecules are of interest given that the response to the histotripsy treatment of pancreatic tumors *in vivo* yielded an inflammatory response that is turned off over time while showing relatively large variations of immune cell proportions within treated tumors. Looking at the release of DNA and peptides as potential DAMPs showed that histotripsy is comparable to the nonthermal ablations cryoablation and IRE (see Fig. 3).

Extracellular DNA is recognized by innate immune system receptors as a DAMP and is not typically found in the absence of damage [44]. In addition, the presence of released proteins increases the probability of the immune system being recruited by damage associated cytokines and antigens. Our hypothesized expectations for the *in vitro* studies were that cryoablation and histotripsy should have similar, more intact DNA, in larger fragments, and more protein released from cells after ablation given that they both ablate cells through immediate lysis [17], [45]. On the other hand, no cell death was observed in untreated cells, also as expected, and therefore, had low levels of protein and DNA. However, IRE induces a delayed cell death, defined as either apoptosis or pyroptosis [15]. DNA fragmentation and protein release or damage are hallmark features of programmed cell death. Similarly, thermal ablation is known to directly denature DNA and protein [16], [46]. Thus, in both these cases, we predicted that the DNA released following ablation would be significantly more fragmented, including significantly smaller and undetectable fragments. Even though IRE does use a delayed cell death mechanism, we did expect to find the higher level of detectable proteins, given that programed cell death does not denature all of the released proteins and previous studies that also showed that IRE can release a significant magnitude of intact proteins [15], [16].

In addition, results from the *in vitro* experiments provide important comparisons between different ablation methods and demonstrate that nonthermal ablation approaches, including histotripsy, lead to significantly increased release of DAMPs and potential antigens in comparison to thermal ablation methods. In comparing the clinical relevance of the differential release of DAMPs and potential antigens that was observed between the ablation modalities *in vitro*, it is important to note that additional *in vivo* studies will be needed to further study the differences between each ablation modality. Similarly, additional *in vivo* studies will be needed to further investigate the role of treatment dose in immune system activation for each of the ablation modalities. For instance, it is not clear whether the partial ablations generated in our *in vitro* studies will be representative of a partial or incomplete ablation in a clinical setting. For the thermal ablation, cryoablation, and IRE samples treated at partial-ablation doses in this study *in vitro*, all of the cells in suspension were treated at a reduced magnitude. In contrast, since histotripsy is a binary treatment that requires a bubble cloud to be formed, once a distinct pressure threshold has been exceeded [47]-[50], the histotripsy partial ablation resulted in only a portion of the cells being exposed to a full amplitude histotripsy bubble cloud, whereas the remaining cells received no treatment. When comparing these *in vitro* results from cell suspensions to *in vivo* ablations, it is important to remember that these differences in how a partial ablation versus a full ablation is acquired may result in different responses. As a result, controlled *in vivo* studies comparing the effects of different ablation modalities and different treatment doses on the potential immunological benefits should be conducted. In addition to comparing the differences between partial and complete ablation, these future studies should investigate the potential risks of overtreatment that could potentially reduce immunological benefits. For instance, in prior work designing IRE protocols, users have outlined specific treatment guidelines to avoid excessive Joule heating to capitalize on the therapeutic benefits of the nonthermal IRE ablation [34]. Similarly, samples overtreated with thermal ablation modalities and cryoablation face an increased rate of macromolecule breakdown and protein denaturation compared to nonthermal [16]. Future studies are needed to determine any effects of overtreatment with histotripsy and to determine the optimal histotripsy treatment strategies for maximizing immunological benefits.

Given that histotripsy’s mechanism of ablation is the mechanical lysis of cells and that, *in vitro*, we found the increased release of DNA and proteins (see Fig. 3), the determination that DAMP signaling pathways [see Fig. 5(f)] were activated *in vivo* was not surprising. Although we did not appreciate histological changes in immune cell infiltration on a microscopic level, gene expression analysis suggested changes in immune cell signaling pathways, which led us to assess immune cell populations via flow cytometry, a more sensitive indicator. Here, we were able to identify specific pathways modulated by histotripsy, including HMGB1, IL-6, Interferon, TEC Kinase, JAK/STAT, and PPAR pathway signaling. These pathways are consistent with responses to trauma or physical damage [51]-[53]. As time passes after trauma, these signaling pathways become repressed to control the immune response. One way by which this is done is through the PPAR pathway, which can inhibit the downstream production of cytokines from the HMGB1 and NF-*κ*B pathways [54]. The activation of the PPAR pathway can also increase healing effects by stimulating the production of VEGF [55], found in our study to be significantly increased in treated survival group tumors (see Fig. 5(f), and see Table I in the Supplementary Material). For healing, the production of VEGF is needed to reestablish healthy vasculature; however, high levels of VEGF within a tumor have been established to be tumorigenic and correlates with poor patient outcomes [56]. Histotripsy increasing the activation of the PPAR pathway and increasing the levels of VEGF expression did not promote negative outcomes for the animals in our study but could be targets of future adjuvants to extend the early inflammatory tumor microenvironment.

At two weeks posthistotripsy ablation, there was a significant decrease in macrophages (see Fig. 6) and Treg cells (see Fig. 7). Significant decreases in the expression of cytokines are associated with tumor-associated macrophages (TAMs), including IL-4, IL-10 CCL-2, CCL-22, and CXCl-12 (see Table I in the Supplementary Material), and suggest that the reduction in macrophages within the treated tumors could be indicative of a decrease in TAMs [57], [58]. A decrease in Treg cells with a potential decrease in TAMs could indicate additional access of histotripsy immunomodulation of the tumor microenvironment to being less anti-inflammatory. This could be a potential target for enhancement with adjuvant therapies [57], [59]. Overall, these changes to immune cell populations should be further analyzed in future studies to determine the full extent of changes to subpopulations, such as changes to M1/M2/TAM macrophages instead of basic populations shown here.

Early studies investigating the immunological effects of histotripsy compared the effects of acoustic cavitation to acoustic heating with thermal HIFU. One study using a subcutaneous colon adenocarcinoma mouse model showed that histotripsy is capable of stimulating CD11c+ cells within the tumors more than thermal HIFU [26]. It has also been shown that histotripsy of murine melanoma can better stimulate an immune response with a decrease in metastasis in the weeks following treatment compared to HIFU, suggesting the involvement of antitumor lymphocytes [27]. More recent studies have further established the proinflammatory local and systemic effects of histotripsy on the immune response in melanoma, neuroblastoma, hepatocellular carcinoma, and renal cell carcinoma [22]-[24]. This study adds to this knowledge by establishing a framework for the immune response to histotripsy ablation of pancreatic cancer. The response reestablished here is similar to other studies. However, given that the Pan02 tumors are known to be poorly immunogenic [60], it was not surprising that the local changes in immune cell populations, while significant at points (see Figs. 7 and 8) were not as prominent of a profile shift as what has been reported in other tumors types. Using the Pan02 model, a prior study with IRE showed that subcutaneous tumors did not have a strong change in immune cell populations within the tumor as orthotopic tumors [61]. Similar to the current work, this prior study showed data that the non-thermal ablation of the subcutaneous pancreatic tumors can shift the tumor microenvironment to being more pro-inflammatory [61].

The results of this study provide evidence that histotripsy can ablate subcutaneous pancreatic tumors and stimulate a local immune response. This study builds upon previous studies utilizing histotripsy for other tumor types [20], [22]-[24] and shows a potential immune profile for pancreatic tumors after histotripsy ablation. Overall, the results of this work provide a baseline expectation of the response of pancreatic tumors to histotripsy, which will help for planning future orthotopic studies.

## V. Conclusion

This study demonstrates the feasibility of using histotripsy for pancreatic cancer ablation and defines mechanisms associated with innate immune system activation following treatment. Further work is needed to establish the mechanisms behind the immunomodulation of the tumor microenvironment and its systemic effects.

## Supplementary Material

Supplementary Material

## Figures and Tables

**TABLE I:** Subject Numbers per Experimental Group

		Immediate Group	Acute Group	Chronic Group	Flow Cytometry
No Treatment	No Tumor	-	3	3	-
	Tumor	3	7	7	4/time point
Histotripsy Treated Tumors		3	7	7	4/time point

**TABLE II:** Flow Cytometry Immune Cell Markers

IMMUNE CELL TYPE	IDENTIFYING MARKERS
Macrophages (MO)	CD45+ CD11c+ F4/80+ Ly6C−
Inflammatory Dendritic Cells (iDCs)	CD45+ CD11c+ F4/80− Ly6C+
Classic Dendritic Cells (cDCs)	CD45+ CD11c+ F4/80− Ly6C−
Monocytic Myeloid Derived Suppressor Cells (M-MDSC)	CD45+ CD11c− Ly6C+ Ly6G−
Granulocytes	CD45+ CD11c− Ly6C− Ly6G+
CD8+ T cells	CD45+ CD3+ CD4− CD8+
CD4+ T cells	CD45+ CD3+ CD4+ CD8−
T Regulatory Cells (Treg cells)	CD45+ CD3+ CD4+ CD8− FoxP3+
T Helper Cells (Th cells)	CD45+ CD3+ CD4+ CD8− FoxP3−
