# Supplementary material for: Histotripsy Ablation Alters the Tumor Microenvironment and Promotes Immune System Activation in a Subcutaneous Model of Pancreatic Cancer
Source: IEEE Trans Ultrason Ferroelectr Freq Control. Author manuscript; Available in PMC 2022 Jul 19. (PMC9295194; doi:10.1109/TUFFC.2021.3078094)

Supplemental Table 1

Symbol	Unigene	Refseq	Fold Regulation			
			24 Hours		Survival	
			Untreated	Treated	Untreated	Treated
Ackr3	Mm.6522	NM_007722	1.00	1.11	1.00	1.00
Aicda	Mm.391503	NM_009645	1.00	1.15	1.00	**-4.44**
Bcl2	Mm.257460	NM_009741	1.00	-1.01	1.00	**-2.06**
Bcl2l1	Mm.238213	NM_009743	1.00	1.00	1.00	**-4.01**
Ccl2	Mm.290320	NM_011333	1.00	**-2.10**	1.00	-1.00
Ccl20	Mm.116739	NM_016960	1.00	1.11	1.00	**-2.01**
Ccl22	Mm.12895	NM_009137	1.00	1.14	1.00	**-34.85**
Ccl28	Mm.143745	NM_020279	1.00	1.08	1.00	**-2.34**
Ccl4	Mm.244263	NM_013652	1.00	**-3.58**	1.00	-1.94
Ccl5	Mm.284248	NM_013653	1.00	1.07	1.00	**-4.04**
Ccr1	Mm.274927	NM_009912	1.00	-1.00	1.00	-1.00
Ccr10	Mm.8021	NM_007721	1.00	1.11	1.00	**-7.56**
Ccr2	Mm.6272	NM_009915	1.00	1.10	1.00	**-9.73**
Ccr4	Mm.1337	NM_009916	1.00	1.16	1.00	**-2.41**
Ccr5	Mm.14302	NM_009917	1.00	-1.90	1.00	-1.75
Ccr7	Mm.2932	NM_007719	1.00	**4.17**	1.00	**-2.45**
Ccr9	Mm.440604	NM_009913	1.00	1.11	1.00	**-7.84**
Cd274	Mm.245363	NM_021893	1.00	**-3.79**	1.00	**-2.01**
Csf1	Mm.795	NM_007778	1.00	1.02	1.00	**2.23**
Csf2	Mm.4922	NM_009969	1.00	-1.94	1.00	**-2.16**
Csf3	Mm.1238	NM_009971	1.00	-1.11	1.00	**-4.33**
Ctla4	Mm.390	NM_009843	1.00	-1.04	1.00	**-9.22**
Cxcl1	Mm.21013	NM_008176	1.00	1.09	1.00	**-7.46**
Cxcl10	Mm.877	NM_021274	1.00	**-4.08**	1.00	**-4.00**
Cxcl11	Mm.131723	NM_019494	1.00	**-3.55**	1.00	**-16.98**
Cxcl12	Mm.303231	NM_021704	1.00	1.04	1.00	1.11
Cxcl2	Mm.4979	NM_009140	1.00	-1.78	1.00	1.03
Cxcl5	Mm.4660	NM_009141	1.00	-1.80	1.00	**-34.96**
Cxcl9	Mm.766	NM_008599	1.00	**4.05**	1.00	**-2.03**
Cxcr1	Mm.337035	NM_178241	1.00	-1.02	1.00	**-8.55**
Cxcr2	Mm.234466	NM_009909	1.00	1.11	1.00	**-8.46**
Cxcr3	Mm.12876	NM_009910	1.00	1.11	1.00	**-2.03**
Cxcr4	Mm.1401	NM_009911	1.00	**4.15**	1.00	**-8.68**
Cxcr5	Mm.6246	NM_007551	1.00	1.15	1.00	**-2.42**
Egf	Mm.252481	NM_010113	1.00	1.19	1.00	**-134.73**
Egfr	Mm.439882	NM_007912	1.00	**-3.53**	1.00	1.00
Fasl	Mm.3355	NM_010177	1.00	1.11	1.00	**-17.21**
Foxp3	Mm.182291	NM_054039	1.00	1.11	1.00	**-8.41**
Gbp2b	Mm.457978	NM_010259	1.00	1.15	1.00	**-4.40**
Gzma	Mm.15510	NM_010370	1.00	1.25	1.00	**-9.28**
Gzmb	Mm.14874	NM_013542	1.00	**4.10**	1.00	**-8.70**
H2-D1	Mm.439675	NM_010380	1.00	-1.80	1.00	**3.99**
H2-K1	Mm.466882	NM_001001892	1.00	-1.88	1.00	1.99
Hif1a	Mm.3879	NM_001313919	1.00	1.18	1.00	**-2.10**
Ido1	Mm.392	NM_008324	1.00	1.11	1.00	**-4.75**
Ifng	Mm.240327	NM_008337	1.00	1.11	1.00	1.10
Igf1	Mm.268521	NM_010512	1.00	-1.95	1.00	-1.97
Il10	Mm.874	NM_010548	1.00	1.11	1.00	**-8.66**
Il12a	Mm.103783	NM_008351	1.00	1.11	1.00	**-8.62**
Il12b	Mm.239707	NM_001303244	1.00	**-3.59**	1.00	**-8.47**
Il13	Mm.1284	NM_008355	1.00	**4.11**	1.00	**-17.44**
Il15	Mm.4392	NM_008357	1.00	1.05	1.00	**-4.00**
Il17a	Mm.5419	NM_010552	1.00	1.10	1.00	**4.16**
Il1a	Mm.15534	NM_010554	1.00	-1.99	1.00	**-8.61**
Il1b	Mm.222830	NM_008361	1.00	1.10	1.00	**-2.03**
Il1r1	Mm.896	NM_008362	1.00	1.11	1.00	**-4.03**
Il2	Mm.14190	NM_008366	1.00	1.22	1.00	**-4.67**
Il22	Mm.103585	NM_016971	1.00	**4.04**	1.00	**-2.16**
Il23a	Mm.125482	NM_031252	1.00	1.07	1.00	**-4.99**
Il4	Mm.276360	NM_021283	1.00	1.15	1.00	**-9.07**
Il5	Mm.4461	NM_010558	1.00	**4.14**	1.00	**-2.20**
Il6	Mm.1019	NM_001314054	1.00	1.11	1.00	**-8.77**
Irf1	Mm.105218	NM_008390	1.00	1.11	1.00	**4.07**
Kitl	Mm.45124	NM_013598	1.00	1.12	1.00	1.95
Mif	Mm.2326	NM_010798	1.00	**-7.15**	1.00	**31.53**
Myc	Mm.2444	NM_010849	1.00	1.12	1.00	**-8.10**
Myd88	Mm.213003	NM_010851	1.00	**-3.33**	1.00	**-2.21**
Nfkb1	Mm.256765	NM_008689	1.00	1.11	1.00	1.07
Nos2	Mm.2893	NM_001313921	1.00	1.07	1.00	**-2.07**
Pdcd1	Mm.5024	NM_008798	1.00	1.14	1.00	**-4.05**
Ptgs2	Mm.292547	NM_011198	1.00	1.03	1.00	-1.02
Spp1	Mm.288474	NM_009263	1.00	-1.79	1.00	**64.77**
Stat1	Mm.277406	NM_009283	1.00	1.11	1.00	1.05
Stat3	Mm.473190	NM_011486	1.00	1.11	1.00	1.98
Tgfb1	Mm.248380	NM_011577	1.00	1.11	1.00	**8.10**
Tlr2	Mm.87596	NM_011905	1.00	-1.04	1.00	1.06
Tlr3	Mm.33874	NM_126166	1.00	1.11	1.00	**-8.77**
Tlr4	Mm.38049	NM_021297	1.00	1.06	1.00	**-2.36**
Tlr7	Mm.23979	NM_133211	1.00	1.21	1.00	**-2.26**
Tlr9	Mm.44889	NM_031178	1.00	1.28	1.00	**-4.05**
Tnf	Mm.1293	NM_013693	1.00	1.14	1.00	**-2.04**
Tnfsf10	Mm.1062	NM_009425	1.00	1.16	1.00	**-9.39**
Trp53	Mm.222	NM_011640	1.00	1.16	1.00	-1.03
Vegfa	Mm.282184	NM_009505	1.00	1.12	1.00	**15.78**
Actb	Mm.328431	NM_007393	1.00	**-3.58**	1.00	**63.90**
B2m	Mm.163	NM_009735	1.00	-1.78	1.00	**-16.62**
Gapdh	Mm.309092	NM_008084	1.00	1.13	1.00	**-4.13**
Gusb	Mm.3317	NM_010368	1.00	1.00	1.00	1.00
Hsp90ab1	Mm.2180	NM_008302	1.00	**2.17**	1.00	1.97

Supplemental Figure 1

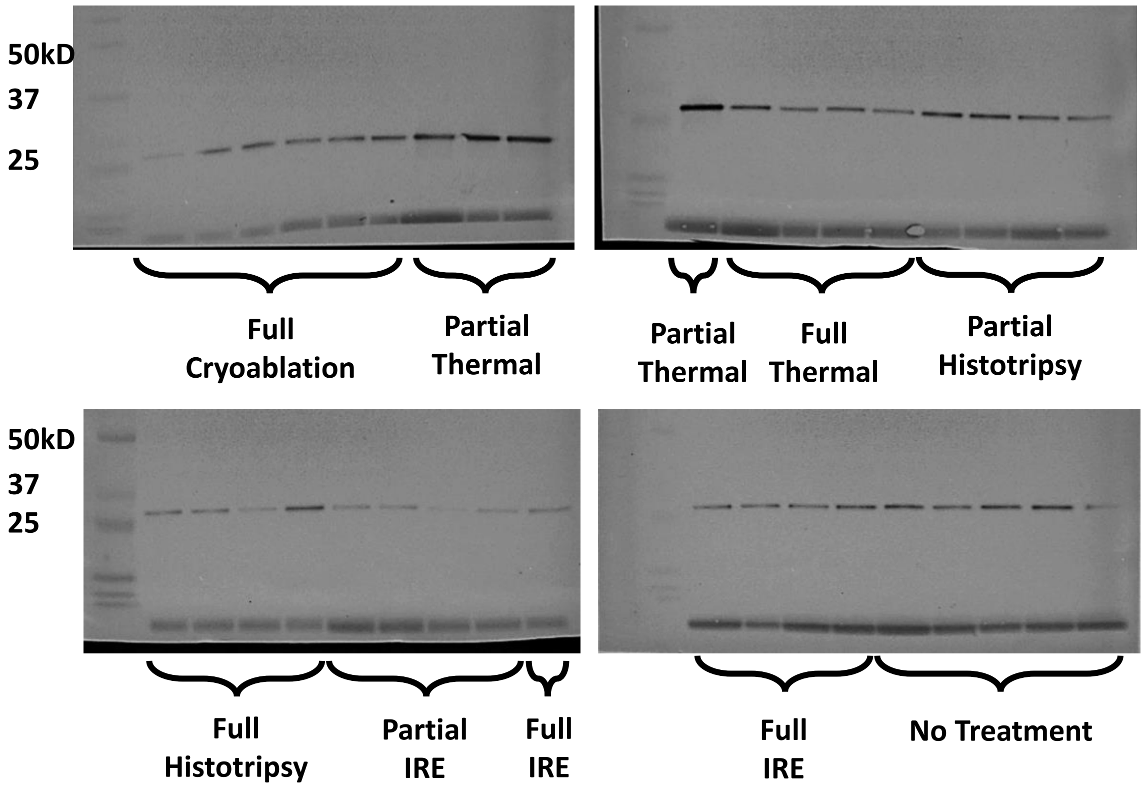

Supplement: Supplementary Material [file NIHMS1821486-supplement-Supplementary_Material.pdf]
